# Combined morphology and radiomics of intravoxel incoherent movement as a predictive model for the pathologic complete response before neoadjuvant chemotherapy in patients with breast cancer

**DOI:** 10.3389/fonc.2025.1452128

**Published:** 2025-02-11

**Authors:** Yunyan Zheng, Hui Zhang, Huijian Chen, Yang Song, Ping Lu, Mingping Ma, Mengbo Lin, Muzhen He

**Affiliations:** ^1^ Shengli Clinical College of Fujian Medical University & Department of Radiology, Fujian Provincial Hospital, Fuzhou University Affiliated Provincial Hospital, Fuzhou, China; ^2^ Shengli Clinical College of Fujian Medical University & Department of Breast Surgery, Fujian Provincial Hospital, Fuzhou University Affiliated Provincial Hospital, Fuzhou, China; ^3^ MR Research Collaboration Team, Siemens Healthineers Ltd., Shanghai, China; ^4^ School of Medical Imaging, Fujian Medical University, Fuzhou, China

**Keywords:** intravoxel incoherent motion (IVIM), breast cancer, neoadjuvant chemotherapy (NACT), radiomics, pathologic complete response (pCR)

## Abstract

**Background:**

To develop a predictive model using baseline imaging of morphology and radiomics derived from intravoxel incoherent motion diffusion-weighted imaging (IVIM-DWI) to determine the pathologic complete response (pCR) to neoadjuvant chemotherapy (NACT) in breast cancer patients.

**Methods:**

A total of 265 patients who underwent 3.0 T MRI scans before NACT were examined. Among them, 113 female patients with stage II–III breast cancer were included. The training data set consisted of 79 patients (31/48=pCR/Non-PCR, npCR), while the remaining 34 cases formed the validation cohort (13/21=pCR/npCR). Radiomics and conventional magnetic resonance imaging features analysis were performed. To build a nomogram model that integrates the radiomics signature and conventional imaging, a logistic regression method was employed. The performance evaluation of the nomogram involved the area under the receiver operating characteristic curve (AUC), a decision curve analysis, and the calibration slope.

**Results:**

In an assessment for predicting pCR, the radiomics model displayed an AUC of 0.778 and 0.703 for the training and testing cohorts, respectively. Conversely, the morphology model exhibited an AUC of 0.721 and 0.795 for the training and testing cohorts, respectively. The nomogram displayed superior predictive discrimination with an AUC of 0.862 for the training cohort and 0.861 for the testing cohort. Decision curve analyses indicated that the nomogram provided the highest clinical net benefit.

**Conclusion:**

Performing a nomogram consisting of integrated morphology and radiomics assessment using IVIM-DWI before NACT enables effective prediction of pCR in breast cancer. This predictive model therefore can facilitate medical professionals in making individualized treatment decisions.

## Introduction

Breast cancer accounts for approximately 30% of female cancers and has a mortality-to-incidence ratio of 15%. Medical professionals have established neoadjuvant chemotherapy (NACT) as the primary treatment for patients diagnosed with locally advanced breast cancer (1). NACT aims to minimize the size of the primary tumor and decrease disease staging, thereby facilitating breast conservation (1, 2). Patients who attain pathologic complete response (pCR) following NACT have exhibited reduced rates of distant recurrence and experienced extended periods of disease-free survival (3–5). However, some patients who underwent NACT did not achieve pCR. Previous reports suggest that the rates of pCR in these patients varied from 45% to 6%, which were determined by the NACT regimen employed and the tumor subtype (6–9). Therefore, timely detection of patients who will not respond to NACT would allow them to avoid taking ineffective therapies, while enabling personalized modifications to be made to their treatment plan.

In accordance with the NCCN Clinical Practice Guidelines in Oncology-Breast Cancer (version 2.2022), Magnetic Resonance Imaging (MRI) is the recommended method for evaluating the response of breast cancer to NACT. MRI can offer higher soft-tissue resolution compared to other methods such as mammography and ultrasonography. According to the 2013 edition of the Breast Imaging Report and Data System (BI-RADS), MRI categorizes breast lesions into masses and non-mass enhancement (NME) lesions based on their morphological characteristics. Different tumor subtypes exhibit distinct morphologies on MRI. Previous studies have indicated a correlation between the baseline lesion morphology and the efficacy of NCAT (10). DWI can indirectly assess tissue differentiation and cell membrane integrity without the need for contrast injection. Apparent diffusion coefficient (ADC) values can quantify water proton diffusion in tissues. However, capillary perfusion in the intravascular extracellular space can impact this process. At present, some studies suggest that the baseline ADC value has no predictive value for NACT (11, 12). Another functional MRI technique, known as intravoxel incoherent motion (IVIM)-DWI, enables visualization of molecular diffusion and perfusion occurring in tissues. Moreover, it allows for the quantification of various perfusion parameters, including microvascular perfusion fraction (f) and incoherent perfusion-related microcirculation (D*) within capillary networks, as well as diffusion parameters such as the pure diffusion coefficient (D) within tissues. This technique utilizes multiple high and low b values (13, 14). Some scholars have used quantitative data of the baseline IVIM to predict NACT efficacy, but predictions are inconsistent (15–17).

In 2012, Dutch researcher Lambin et al. (18) initially introduced the concept of radiomics, which has emerged as a valuable tool in the field of oncology. Radiomics enables the transformation of standard digital imaging into quantitative, mineable data, allowing for the noninvasive characterization of tumors and the expression of various tumor properties (19, 20). Breast cancer tumors reveal strong temporal and spatial heterogeneity. Tumor heterogeneity analyses can be conducted using medical imaging techniques such as MRI, which eliminates the need for additional data collection. However, currently, there is no application that includes baseline IVIM radiomics data for predicting the effectiveness of NACT in breast cancer.

This study aims to develop morphology and radiomics models based on IVIM DWI baseline imaging for the purpose of predicting the pCR to NACT in patients with breast cancer.

## Materials and methods

### Study setting and timeframe

**Figure 1:**
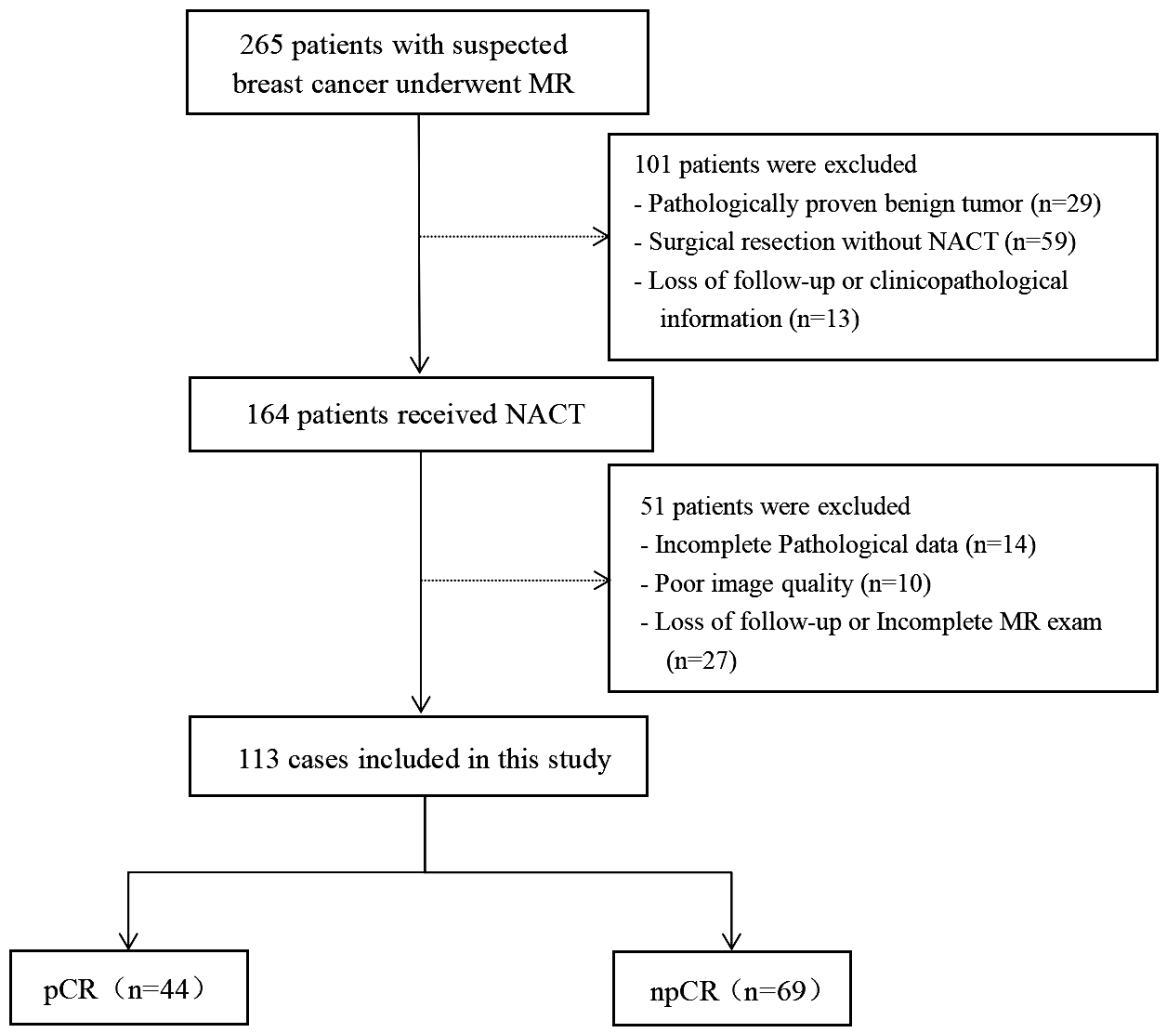
Flowchart depicting case selection and distribution.

This prospective study consecutively enrolled patients with suspected breast cancer who underwent treatment at Fujian Provincial Hospital, between July 2019 and October 2022. The ethics committee of Fujian Provincial Hospital approved the study protocol (approval code: K2021-05-007, May 2019), and all methods in this study were carried out in accordance with relevant guidelines and regulations (21). Written informed consent to participate was obtained from all the patients. The inclusion criteria were: (I) no needle biopsy, radiotherapy, or chemotherapy before MRI examination; (II) availability of complete MRI review data before NAT with good image quality; (III) availability of complete pathological data; and (IV) without multicentric tumor. The process of patient selection and grouping has been illustrated in **Figure 1**.

### Interventions

After the MR examination, patients with suspected breast cancer undergo a needle biopsy of breast lesions to decide on further treatment options. All included patients received NACT, which was formulated according to the institution’s standards (7). Patients received intravenous administration of the chemotherapy regimen every 3 weeks for six to eight cycles. Following that, the patients underwent surgical removal of their tumors and regional lymph nodes.

### Neoadjuvant chemotherapy and histopathologic analysis

Among the 113 included patients, chemotherapy regimens were distributed as (attachment 1). NACT was determined based on the guidelines of the Chinese Society of Clinical Oncology from 2019 to 2022 (21). Following the completion of six to eight cycles of NACT, resected breast tissue and regional lymph nodes obtained from the patient were fixed, paraffin-embedded, and subsequently sliced into thin sections. Hematoxylin and eosin staining was then performed on these biopsy specimens. A pathologist experienced in diagnosing breast tumors was responsible for examining the tissues. The residual condition of the tumor was observed. Based on the 2021 guidelines of the Chinese Society of Clinical Oncology Breast Cancer, pCR is characterized by the absence of invasive carcinoma in the primary breast (with the possibility of ductal carcinoma *in situ*) and in the negative regional lymph node. This criterion can be fulfilled if the primary tumor is categorized as MP (Miller–Payne) grade 5 and shows negative lymph node involvement, or if the residual cancer burden evaluation system assigns a grade of 0.

### Image acquisition and analysis

Magnetic resonance (MR) examinations were conducted using a 3.0T MR scanner (MAGNETOM Prisma, Siemens Healthcare, Erlangen, Germany) equipped with an 18-channel dual-breast-dedicated phase array surface coil. Patients were scanned in the prone position, allowing the breasts to be naturally positioned within the coil. The imaging protocol encompassed the following sequences (**Table 1**). For contrast enhancement, gadopentetate meglumine injection (Magnevist, 0.2 mmol/kg; GE Healthcare) was administrated through the dorsal vein of the hand using a high-pressure syringe at a rate of 1.5–2.0 mL/s. The connecting tube was then flushed with 15–20 mL of normal saline to clear any residual contrast agent.

**Table 1:** The imaging protocol of MR sequences.

Fat saturation T2WI	TR	3739ms	TE	69ms	Thickness	4mm
	Slices	35	Bandwidth	246Hz/Px	FOV	340mm
	FOV phase	100%	Matrix	384×384	Averages	2
	Concatenations	2				
T1WI	TR	6.03ms	TE	2.82ms	Thickness	0.9mm
	Slices	160	Bandwidth	300Hz/Px	FOV	340mm
	FOV phase	100%	Matrix	403×448		
DCE-MRI	TR	4.03ms	TE	1.33ms	Thickness	1.5mm
	Slices	112	Bandwidth	1120Hz/Px	FOV	350mm
	FOV phase	100%	Matrix	259×320	Measurements	36
	Volume	350 mm × 350 mm × 1.5 mm × 112			Scan time	343s
DWI	TR	5700ms	TE	62ms	Thickness	4mm
	Slices	35	Bandwidth	2024Hz/Px	FOV	340mm
	FOV phase	60%	Matrix	114×190		
	B-values	0,30,50,80,120,160,200,500,1000s/mm2				
	Averages	1, 2, 2, 2, 2, 2, 2, 2, 3			Scan time	318s

DCE-MRI, dynamic contrast-enhanced MRI; DWI, diffusion-weighted imaging; FOV, Field of View.

### Tumor segmentation and image feature extraction

**Figure 2:**
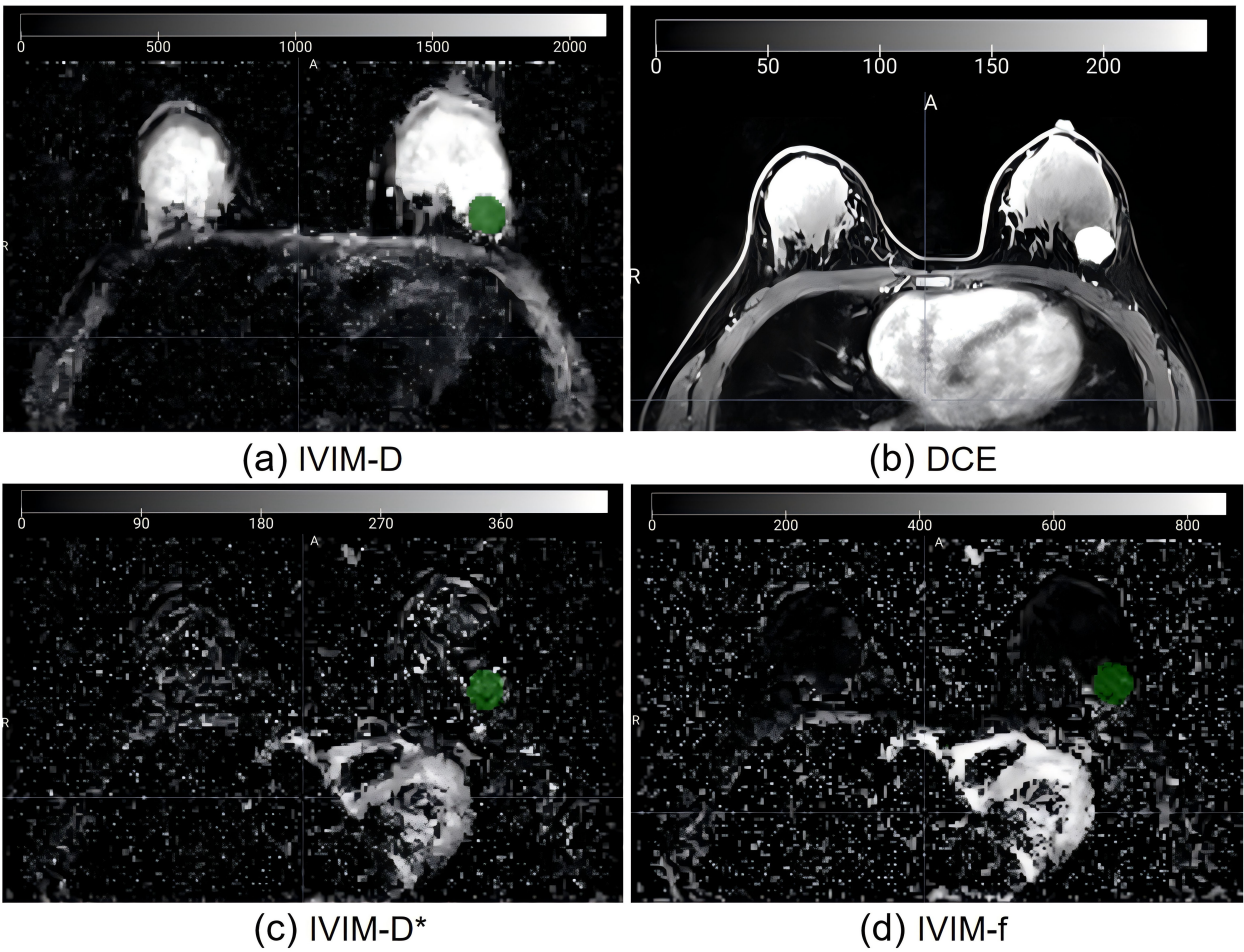
Imaging of a 56-year-old female patient with invasive ductal carcinoma. **(A)** ROI on IVIM-D map refer to the DCE map **(B)** to determine the edge; **(C)** Same ROI on IVIM-D*map; **(D)** Same ROI on IVIM-f map. IVIM, intravoxel incoherent motion; ROI, region of interest; D, pure diffusion coefficient; D*, incoherent perfusion-related microcirculation; f, microvascular perfusion fraction.

**Figure 3:**
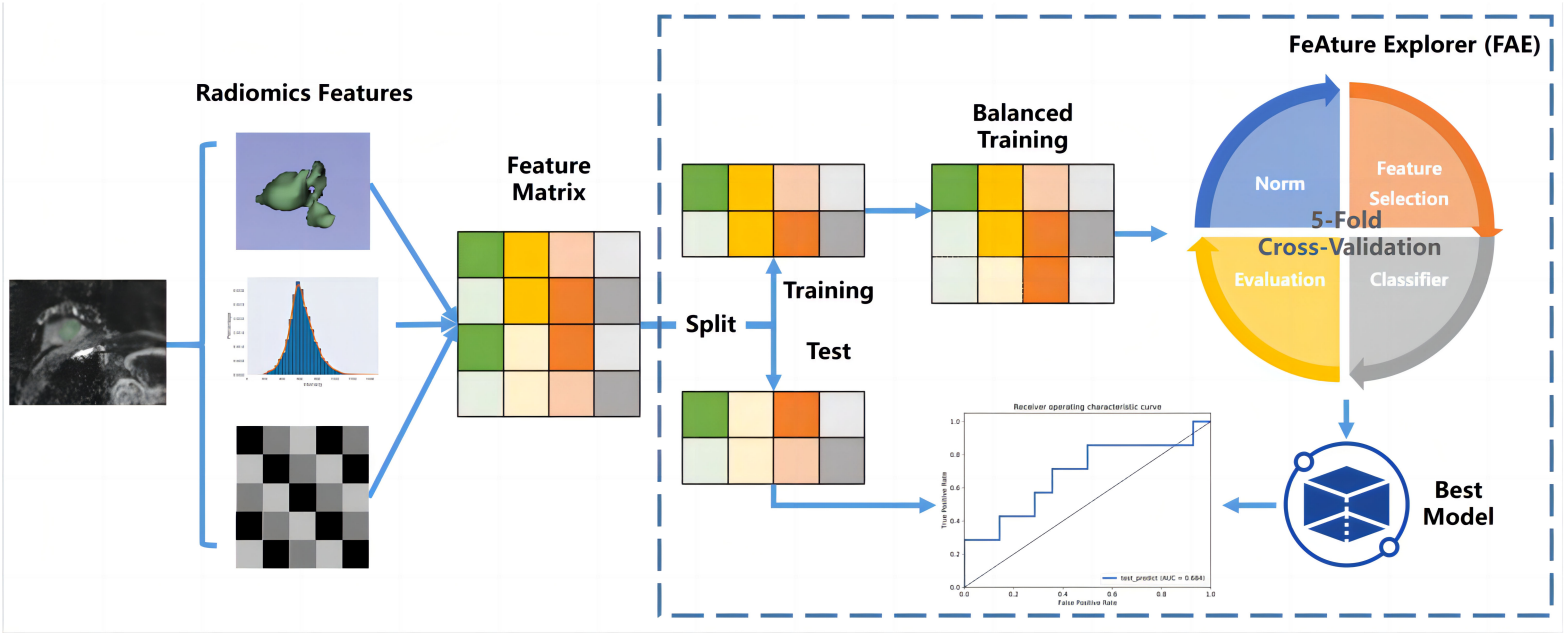
Workflow for image features acquisition.

To obtain IVIM maps, a pixel-by-pixel fitting of DWI data was carried out using a research prototype software called Body Diffusion Toolbox (Siemens Healthcare, Erlangen, Germany). This software generated parameter maps for IVIM, including a perfusion tissue diffusion coefficient (D), a fraction (f), and perfusion-related incoherent microcirculation (D*). The IVIM model, a total of nine b-values (0, 30, 50, 80, 120, 160, 200, 500, and 1000 s/mm^2^) were used for data calculation using the classic two-step calculation method. We set the b-value threshold value as 200 to split. For the two-step fitting method, we first fit the D, and then fixed the D and fit the D* and f. The IVIM images were visualized using 3D Slicer software (version 4.10.2, www.slicer.org) for image segmentation. A single radiologist (M.H.) with 13 years of experience in breast imaging diagnosis conducted the segmentation process. A three-dimensional region of interest (ROI) that included the solid tumor component was drawn on D-maps, and for lesion edges whose contrast were suboptimal, we could refer to the DCE map to determine the edge (**Figure 2**). We ensured that the ROI delineation was performed on the IVIM-D map. **Figure 3** shows the tumor segmentation and image feature extraction. From the f, D, and D* parameter maps, we extracted the radiomics features including shape-based, first-order, and texture features. An additional radiologist (Y.Z.) with 5 years of experience in breast imaging independently segmented 30 randomly chosen tumors from the training set. The interobserver reproducibility was evaluated using a two-way random absolute agreement intraclass correlation coefficient (ICC). ICC values varied from 0 to 1, with interpretations as follows: ICC above 0.75 indicated good consistency; ICC between 0.50 and 0.75 pointed to general consistency; and ICC less than 0.50 highlighted poor consistency.

Features with ICCs less than 0.75 were excluded (22–24), the residual features were recognized as stable. During the model-building process on the training data cohort, the first step was to address data imbalance. To achieve a balanced distribution of positive and negative samples, we up-sampled the training data cohort by randomly repeating cases. Subsequently, we performed feature normalization by mean normalization. As the dimensionality of the feature space exceeded the number of cases, we employed principle component analysis (PCA) on the training feature matrix to describe features using the eigenvector. This transformation resulted in independent feature vectors. Then, we used a Gaussian process classifier with recursive feature elimination to determine the final model, specifically selecting relevant features. To determine the optimal number of features and corresponding hyper-parameters, we utilized a 5-fold cross-validation on the training data cohort.

To evaluate the model’s performance, we used receiver operating characteristic (ROC) curve analysis. The quantification of performance was based on the area under the ROC curve (AUC). To determine accuracy, sensitivity, specificity, positive predictive value, and negative predictive value, we utilized a cutoff value that maximized the Youden index. Furthermore, to estimate the 95% confidence interval, we employed a bootstrap with 1,000 samples. All of these analyses were carried out using FeAture Explorer (FAE, V 0.5.5) (25) (**Supplementary Material S1**).

### Statistical methods

#### Predictive ability of the morphology model

Breast lesions were evaluated for conventional MR imaging signs based on the 2013 BI-RADS Magnetic Resonance Imaging guidelines by two physicians with 14 years and 3 years of experience in breast imaging diagnosis, respectively. Any disagreements between the 2 physicians were discussed until consensus was achieved. We analyzed 6 conventional MRI features for each patient: masses or non-mass enhancement, tumor shape, tumor margin, internal enhancement characteristics, NME distribution, and NME internal enhancement pattern. Categorical variables were presented as frequencies (percentages), and the differences between groups were determined using Pearson’s Chi-squared test or the continuous-corrected Chi-squared test.

#### Predictive ability of the nomogram model

A logistic regression model was utilized to build a nomogram model to integrate the radiomics signature and conventional imaging. The combined model’s predictive ability was evaluated using ROC curves and various classification measures, including AUC, sensitivity, specificity, and accuracy. Calibration plots were conducted to assess nomogram performance by comparing observed probabilities with nomogram-predicted probabilities. Additionally, decision curve analysis was employed, plotting the net benefit rate against the threshold of high risk. Morphology, radiology, and the combined model were analyzed to predict the net benefit of the pCR.

### Follow-up

Patients were followed up after two to three cycles of chemotherapy and upon completion of chemotherapy. During these follow-up visits, the patients underwent MR scanning to evaluate the status of their tumors. The patients’ tumors were evaluated for pCR status after they were surgically removed.

## Results

### Participants characteristics

The study consisted of 113 female patients diagnosed with stage II–III breast cancer. For the training data cohort, 79 cases were randomly selected (31/48 = pCR/npCR), while the remaining 34 cases formed the independent testing data cohort (13/21 = pCR/npCR). The mean age of the patients was 52.6 ± 10.2 years (range, 25–75). We collected clinicopathological data prior to the administration of NACT. Among the cases, 107 were diagnosed with invasive ductal carcinoma, four with invasive lobular carcinoma, and two with metaplastic carcinoma. A detailed summary of this information can be found in **Table 2**.

**Table 2:** Characteristics of patients in the training and testing cohorts.

Characteristic	Training cohort		*P* value	Testing cohort		*P* value
	pCR	npCR		pCR	npCR	
Age (years), Mean ± SD	51.9 ± 9.7	53.2 ± 10.8	0.579	52.1 ± 10.9	53.5 ± 8.9	0.764
Histological subtype (%)			0.942			1.000
Ductal	30 (30/79,38.0%)	45 (45/79,57.0%)		12 (12/34,35.3%)	20 (20/34,58.9%)	
Others	1 (1/79,1.3%)	3 (3/79,3.8%)		1 (1/34,3.0%)	1 (1/34,3.0%)	
HR status (%)			0.001			0.039
Positive	8 (8/79,10.1%)	30 (30/79,38.0%)		3 (3/34,8.9%)	13 (13/34,38.2%)	
Negative	23 (23/79,29.1%)	18 (18/79,22.8%)		10 (10/34,29.4%)	8 (8/34,23.5%)	
HER2 status (%)			0.008			0.491
Positive	23 (23/79,29.1%)	21 (21/79,26.6%)		7 (7/34,20.6%)	14 (14/34,41.2%)	
Negative	8 (8/79,10.1%)	27 (27/79,34.1%)		6 (6/34,17.6%)	7 (7/34,20.6%)	
Ki67 (%)			0.040			0.144
High	28 (28/79,35.4%)	34 (34/79,43.0%)		13 (13/34,38.2%)	17 (17/34,50.0%)	
Low	3 (3/79,3.8%)	14 (14/79,17.7%)		0 (0/34,0%)	4 (4/34,11.8%)	
Clinical stage (%)			0.811			0.728
II	14 (14/79,17.7%)	23 (23/79,29.1%)		5 (5/34,14.7%)	10 (10/34,29.4%)	
III	17 (17/79,21.5%)	25 (25/79,31.6%)		8 (8/34,23.5%)	11 (11/34,32.4%)	

Definition of Ki67 high: ≥20% of tumor cell nuclei stained for Ki67; HR, hormone receptor; HER2, human epidermal growth factor receptor 2; pCR, pathologic complete response.

### Deviation from the initial management plan

The study excluded patients who deviated from the initial management plan or voluntarily withdrew after starting NACT. A description of these exclusions can be found in **Figure 1**.

### Performance of the radiomics model

**Figure 4:**
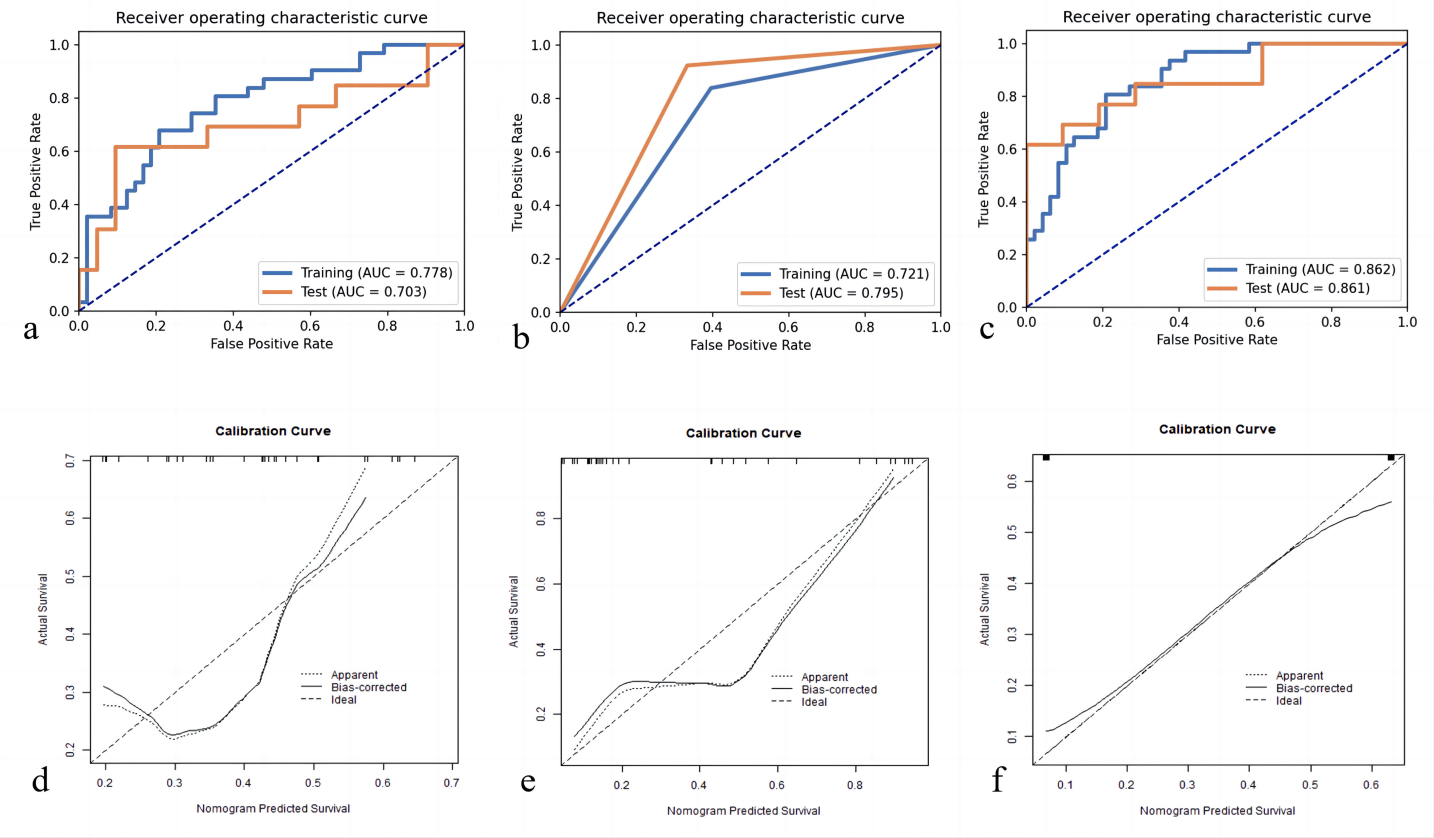
Receiver operating characteristic curves for pCR prediction model: **(A)** Radiomics Model; **(B)** Morphology Model; **(C)** Combined Model. pCR, pathologic complete response; Calibration plot of predicted pCR by the models with actual pCR: **(D)** Radiomics Model; **(E)** Morphology Model; **(F)** Combined Model. pCR, pathologic complete response.

We first extracted 321 features from the f, D, and D* parameter maps. And then we estimated the ICC value for each feature. Finally a total of 278 features remained. The radiomics features were extracted from the original maps and then we used PCA to merge the feature matrix in order to reduce the dimensions of feature space. Our validation process revealed that using only four PCA features in the model resulted in the highest AUC. Specifically, the AUC for the training cohort was 0.778, while the testing cohort exhibited an AUC of 0.703. Clinical statistics for the diagnosis can be found in **Table 3**. **Figure 4** displays the ROC curve of the radiomics model. Moreover, the calibration plots from the radiomics model exhibited a remarkable fit with the ideal curve, with a mean absolute error of 0.07 (**Figure 4**). Furthermore, the predicted pCR demonstrated excellent concordance with the observed pCR.

**Table 3:** Results of ROC curve analysis for predicting pCR using the radiomics model, the morphology model, and the combined model before NACT.

		AUC (95% CI)	Cut-off	Youden Index	Sensitivity	Specificity	Positive predictive value	Negative predictive value
Radiomics Model	Training cohort	0.778 (0.673-0.883)	0.681	0.443	0.839	0.604	0.579	0.853
	Testing cohort	0.703 (0.498-0.908)	0.681	0.590	0.923	0.667	0.632	0.933
Morphology Model	Training cohort	0.721 (0.625-0.817)	0.550	0.437	0.645	0.792	0.667	0.775
	Testing cohort	0.795 (0.667-0.923)	0.550	0.443	0.539	0.905	0.778	0.760
Combined Model	Training cohort	0.861 (0.723-0.999)	0.722	0.615	0.615	1.000	1.000	0.808
	Testing cohort	0.862 (0.784-0.941)	0.474	0.598	0.807	0.792	0.714	0.864

AUC, the area under the receiver operating characteristic curve; NACT, neoadjuvant chemotherapy; pCR, pathologic complete response.

### Performance of the morphology model

Based on the guidelines provided by the American College of Radiology BI-RADS Magnetic Resonance Imaging 2013, a total of 113 lesions were analyzed. Conventional MRI features for each patient were analyzed. Among these, 64 (56.6%) presented as masses, while 49 (43.3%) displayed NME on conventional MR images. Detailed information regarding the conventional MR image model in the training and testing cohorts can be found in **Table 4**. Morphology Model was built using the feature enhancement mode (masses or NME). The model achieved an AUC of 0.721 in the training cohort and 0.795 in the testing cohort, as outlined in **Table 3**. The ROC curve of the conventional MR image mode is shown in **Figure 4**. Moreover, the calibration plots generated from the morphology model displayed a close fit to the ideal curve, with a mean absolute error of 0.059 (**Figure 4**). Additionally, the predicted pCR exhibited strong concordance with the observed pCR.

**Table 4:** Conventional MR signs before NACT of 113 breast cancer patients.

		pCR (n=44)	npCR (n=69)	*t*/*χ²*	*P* value
Size(millimeter)		36.8 ± 8.7	41.8 ± 10.7	0.765	0.145
Masses		38 (38/64, 86.3%)	26 (26/64, 37.6%)	25.927	<0.001
	Shape (%)			0.013	1.000
	Round/Oval	21 (21/64, 32.8%)	14 (14/64, 21.9%)		
	Irregular	17 (17/64, 26.6%)	12 (12/64, 18.8%)		
	Margin (%)			0.657	0.534
	Circumscribed	9 (9/64, 14.1%)	4 (4/64, 6.3%)		
	Not circumscribed	29 (29/64, 45.3%)	22 (22/64, 34.4%)		
	Internal enhancement characteristics (%)			0.623	0.336
	Homogeneous	2 (2/64, 3.1%)	2 (2/64, 3.1%)		
	Heterogeneous	7 (7/64, 10.9%)	6 (6/64, 9.4%)		
	Rim enhancement	29 (29/64, 45.3%)	18 (18/64, 28.1%)		
Non-mass enhancement		6 (6/49, 13.6%)	43 (43/49, 62.3%)		
	Distribution (%)			0.708	0.605
	Focal	0	2 (2/49, 4.1%)		
	Linear/Segmental	3 (3/49, 6.1%)	21 (21/49, 42.9%)		
	Regional/Multiple regions	3 (3/49, 6.1%)	17(17/49, 34.7%)		
	Diffuse	0	3 (3/49, 6.1%)		
	Internal enhancement patterns (%)			1.663	0.255
	Homogeneous	1 (1/49, 2.0%)	3 (3/49, 6.1%)		
	Heterogeneous	2 (2/49, 4.1%)	11 (11/49, 22.4%)		
	Clumped/Clustered ring	3 (3/49, 6.1%)	29 (29/49, 59.2%)		

pCR, pathologic complete response; NACT, neoadjuvant chemotherapy.

### Performance of the morphology model combined with the radiomics signatures

**Table 3** presents the performance of the combined conventional MR image model and radiomics signature model in predicting pCR for breast cancer patients undergoing NACT. **Figure 4** demonstrates the ROC curves for these models in the training and validation cohorts. The AUC values obtained for the training and test cohorts are 0.861 (95%CI: 0.723–0.999) and 0.862 (95%CI: 0.784–0.941), respectively.

**Figure 5:**
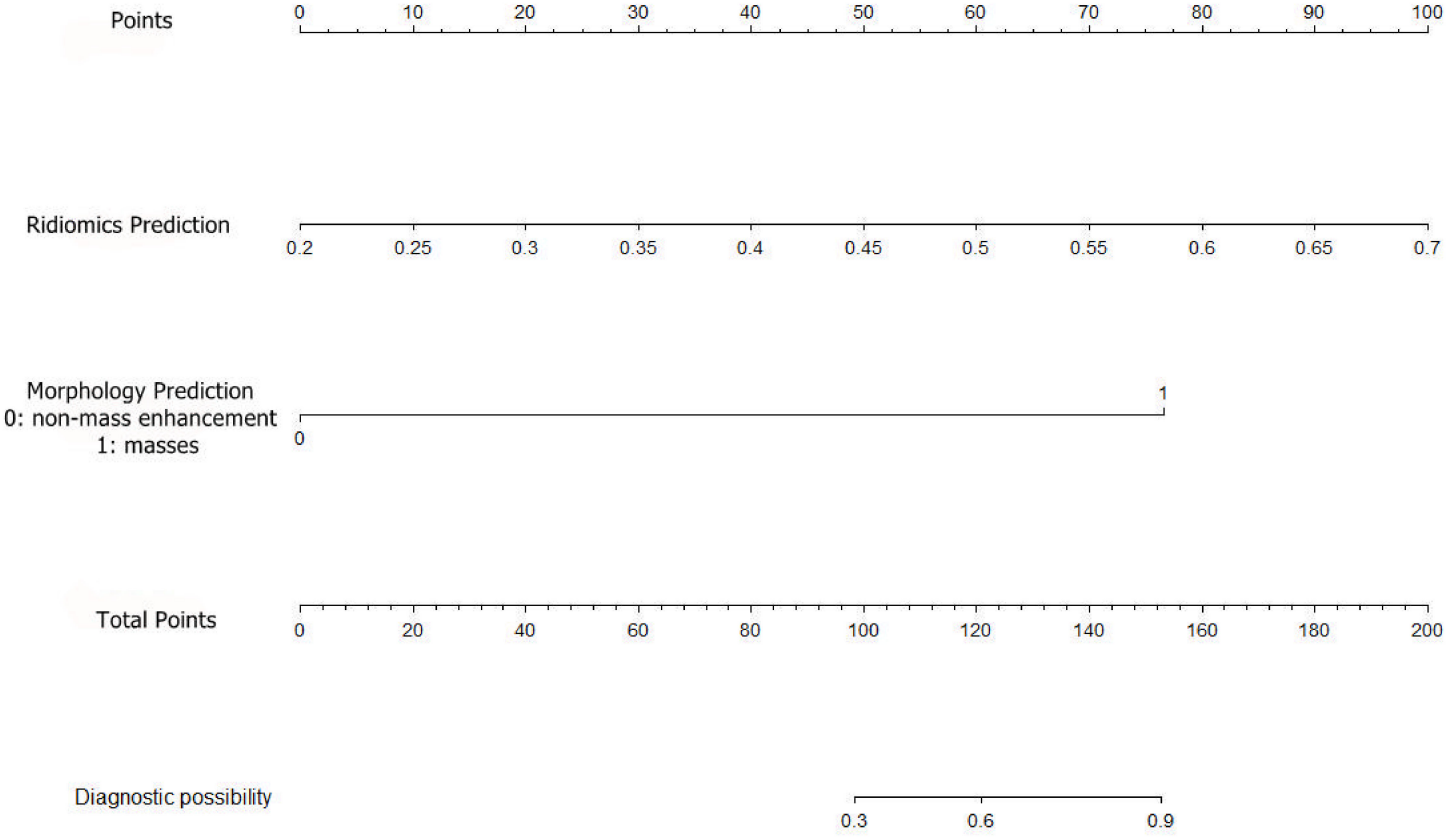
Establishment of a radiomics prediction-morphology prediction-based nomogram for predicting pCR. pCR, pathologic complete response.

**Figure 6:**
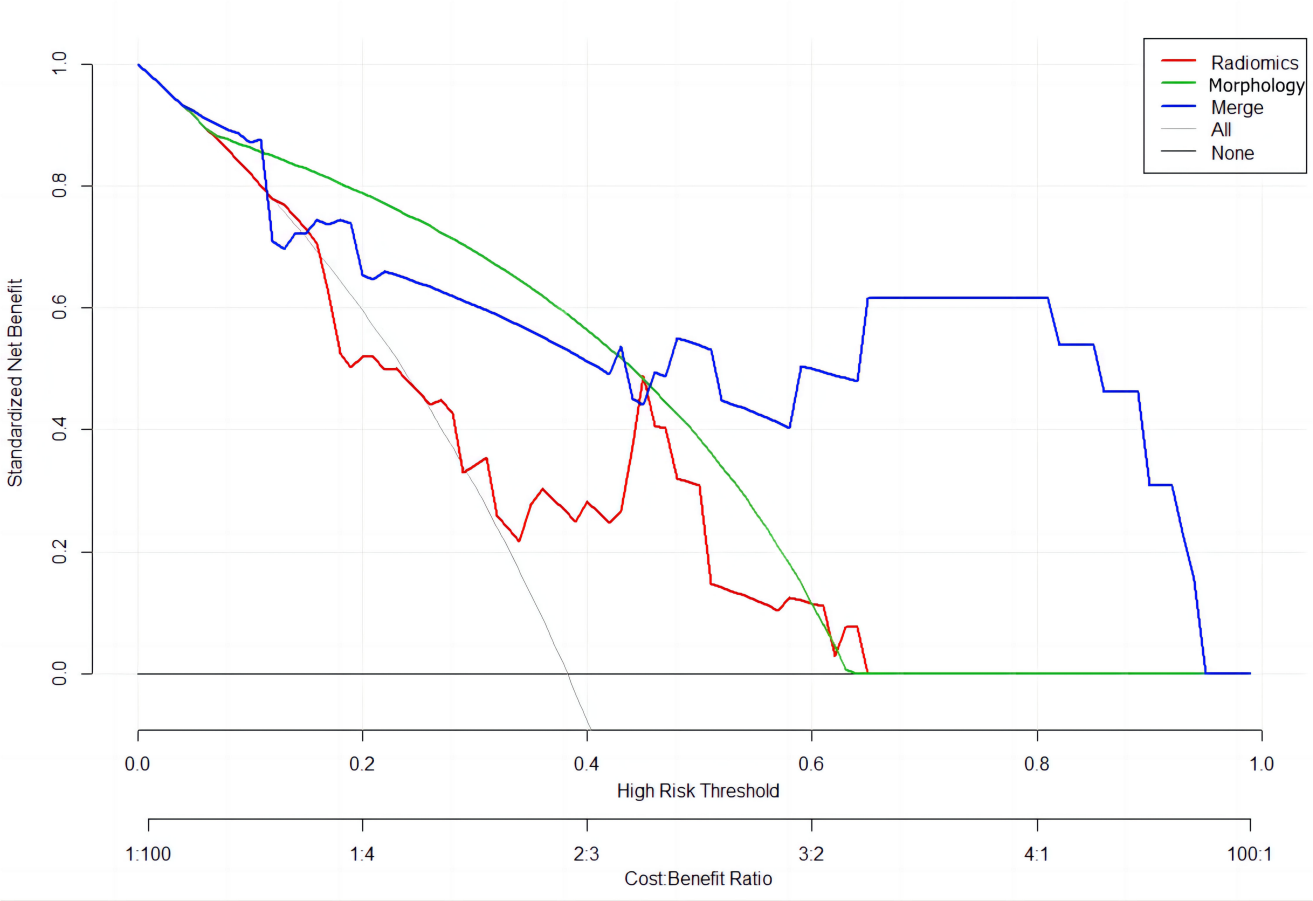
Decision curve analysis for the models’ performance: red line (Radiomics Model); green line (Morphology Model); blue line (Combined Radiomics and Morphology Model).

A combined radiomics and morphology nomogram model was developed (**Figure 5**) to predict the likelihood of achieving pCR. Each predictive factor was assigned a score based on the upper scale line, allowing the total score to be calculated. The calibration plots generated from the morphology model demonstrated good agreement with the ideal curve, yielding a mean absolute error of 0.089 (**Figure 4**). Moreover, the predicted pCR exhibited good concordance with the observed pCR. In the threshold range of 0.5 to 0.9, the combined model provided a greater net benefit for clinical intervention compared to other models (**Figure 6**). Additionally, the decision curve analysis revealed favorable clinical utility of the nomogram.

## Discussion

Precise prediction of post-NACT pCR in breast cancer patients plays a vital role in treatment decision-making and patient prognosis. In the present study, we developed radiomics models of IVIM diffusion-weighted baseline imaging associated with conventional MR images to predict pCR in NCAT-treated breast cancer patients. The prediction model achieved an AUC of 0.862 in the training cohort, while the AUC in the test cohort was 0.861.

Previous studies have indicated the importance of including b-values exceeding 200 s/mm^2^ in the IVIM model (13). In our study, we selected nine b-values, ensuring that seven of them were below 200 s/mm^2^ to accurately capture water molecule diffusion and blood microcirculation perfusion. The IVIM map can reflect both diffusion information and perfusion signals. Tumor uptake of chemotherapy drugs depends on local blood perfusion and capillary permeability. The f value, representing angiogenesis in immature blood vessels and partially reflecting microvascular permeability, may serve as an indicator of the effectiveness of chemotherapy drugs and the likelihood of a favorable response. Therefore, perfusion fraction f could potentially differentiate between pathological responses to NACT before treatment (16). Notably, NACT exhibits enhanced efficacy in biologically aggressive tumors, as these tumors show heightened responsiveness to chemotherapy during their proliferative state. D and D* values demonstrate an inverse correlation with tumor aggressiveness and cell count, making them valuable parameters that can be obtained without the need for contrast medium application (26).

Many studies have indicated that changes in post-NACT IVIM parameters can predict NACT efficacy. Previous research by some investigators has shown that baseline IVIM quantitative parameters before neoadjuvant therapy cannot predict neoadjuvant chemotherapy efficacy (17, 27). In this study, we extracted information from IVIM images using machine learning methods to predict efficacy even before NACT. In addition, the 3D-ROI allows for a more comprehensive assessment of tumor heterogeneity, including the overall information on solid tumors, as compared to the previous 2D-ROI method. This increased predictive efficacy without requiring additional patient examinations. By utilizing volumetric sampling of the entire tumor, sampling bias can be minimized, unlike with the single-section ROI method. Guidelines further endorse this method for evaluating tumor response (28). Some scholars have used DCE-MRI to predict the efficacy of neoadjuvant therapy (29, 30). However, DCE-MRI needs contrast agent injection and the use of different contrast agents and can be influenced by differences in individual circulation. These factors may cause differences in the accuracy of the model among individuals. IVIM MRI, a functional MRI technique, enables the visualization of molecular diffusion and perfusion in tissues and the quantification of specific perfusion parameters, without the need for contrast agent injection. The radiomics model based on relevant parameter maps of IVIM achieved an AUC of 0.778 in the training cohort and 0.703 in the testing cohort, indicating strong predictive capability.

Earlier studies have demonstrated that in breast cancer, the baseline map of NACT reveals pCR more prominently in masses compared to NME (10). Furthermore, luminal tumors (hormone receptor-positive and HER2-negative) are more prone to present as NME or diffuse lesions at baseline and show a multicenter withdrawal pattern after chemotherapy. In contrast, triple-negative and HER2-positive tumors typically appear as discrete masses and display a concentric withdrawal pattern after NACT (31–33). The morphological signs of the baseline map of breast cancer are easy to assess with high agreement among different observers, and morphological prediction of the baseline map has a certain value. When combined with radiomic features, morphological information can improve the predictive efficacy of the model.

### Limitations

We must acknowledge the limitations of our study, including potential influences on the manual image segmentation process due to various objective and subjective factors. Another limitation of our study is the use of PCA for feature reduction. While PCA effectively transforms the original features into a lower-dimensional space to maximize variance, it inherently combines features from all input maps (e.g., D, f, D*), making it challenging to directly attribute the contribution of individual maps to the extracted features. This lack of interpretability may limit the understanding of which specific imaging maps contribute the most to the final radiomic analysis. Future work could explore alternative methods or additional analyses to better interpret feature contributions. Additionally, our model was developed using a single-center cohort without external validation. To enhance confidence in the pCR prediction performance of the model, it is crucial to conduct additional multi-center studies involving larger patient cohorts.

## Conclusion

Through the validation process, the nomogram developed in this study showed good performance, effectively predicting pCR in breast cancer. Our radiomics model using IVIM diffusion-weighted imaging significantly enhances the ability to predict pCR in breast cancer patients undergoing neoadjuvant chemotherapy (NACT). It allows for early identification of treatment efficacy, helping clinicians tailor therapies to individual patients and reduce unnecessary treatments. This model supports more personalized care by providing detailed insights into tumor response and characteristics. It also informs surgical decisions by predicting which patients may benefit from less or more extensive procedures. Future multi-center studies are needed to validate and integrate the model into routine clinical workflows for broader use.

## Data Availability

The raw data supporting the conclusions of this article will be made available by the authors, without undue reservation.
