# Supplementary material for: Combined morphology and radiomics of intravoxel incoherent movement as a predictive model for the pathologic complete response before neoadjuvant chemotherapy in patients with breast cancer
Source: Front Oncol. 2025 Feb 11;15:1452128. doi: 10.3389/fonc.2025.1452128 (PMC11850367; doi:10.3389/fonc.2025.1452128)
Supplement: Supplementary file 2 [file Table1.doc]

The TCbHP regimen (carboplatin, docetaxel, pertuzumab, trastuzumab) was provided to 29 patients; the AC-THP regimen (trastuzumab, cyclophosphamide, paclitaxel, epirubicin, pertuzumab) was provided to 20 patients; the AC-T regimen (epirubicin, cyclophosphamide, paclitaxel) was provided to 19 patients; the TAC regimen (paclitaxel or docetaxel, epirubicin, cyclophosphamide) was provided to 18 patients; the AC regimen (epirubicin, cyclophosphamide) was provided to 16 patients; the AC-TP regimen (cyclophosphamide, epirubicin, paclitaxel, carboplatin) was provided to 4 patients; the TP regimen (paclitaxel, carboplatin) was provided to 3 patients; the AC-H regimen (epirubicin, cyclophosphamide, trastuzumab) was provided to 3 patients; the THP regimen (trastuzumab, paclitaxel, pertuzumab) was provided to 1 patient; and the AT regimen (epirubicin, paclitaxel) was provided to 1 patient.
